# Corrosion Behavior of SMA490BW Steel and Welded Joints for High-Speed Trains in Atmospheric Environments

**DOI:** 10.3390/ma12183043

**Published:** 2019-09-19

**Authors:** Xiangyang Wu, Zhiyi Zhang, Weichuang Qi, Renyong Tian, Shiming Huang, Chunyuan Shi

**Affiliations:** 1 CRRC Sifang Co. Ltd., Qingdao 266111, China; 2 School of Materials Science and Engineering, Dalian Jiaotong University, Dalian 116028, China

**Keywords:** bogie, SMA490BW, corrosion behavior, atmospheric environment, corrosion weight loss rate, potentiodynamic polarization curve

## Abstract

Currently, high-speed trains work under various atmospheric environments, and the bogie as a key component suffers serious corrosion. To investigate the corrosion behavior of bogies in industrial atmospheric environments, the periodic immersion wet/dry cyclic corrosion test for SMA490BW steel and automatic metal active gas (MAG) welded joints used for bogies was conducted in the present work. Corrosion weight loss rate, structure, and composition of rust layers as well as electrochemistry parameters were investigated. The results showed that the corrosion weight loss rate decreased with increasing corrosion time; furthermore, the corrosion weight loss rate of the welded joints was lower than that of SMA490BW steel. The XRD results showed that the rust layers formed on SMA490BW steel and its welded joints were mainly composed of α-FeOOH, γ-FeOOH, Fe_2_O_3_, and Fe_3_O_4_. The observation of surface morphology indicated that the rust layers of the welded joints were much denser and had a much finer microstructure compared with those of SMA490BW steel. After corrosion for 150 h, the corrosion potential of the welded joints with rust layers was higher than that of SMA490BW steel. In short, the welded joints exhibited better corrosion resistance than SMA490BW steel because of the higher content of alloy elements, as shown in this work.

## 1. Introduction

The bogie, as one of the key components of high-speed trains, operates with the functions of load bearing, guidance, and propulsion, and its performance affects the running quality and security of high-speed trains directly [1,2]. High-speed trains work under various atmospheric environments, and the bogie’s surface suffers different degrees of corrosion. A corrosion status survey of high-speed trains in commission showed that the bogie was affected by serious corrosion, and that the corrosion reaction would continue in a corrosive environment [3,4,5]. More importantly, the bogie’s bearing strength is reduced due to the corrosion of the bogie, especially of its welded joints. This can lead to a decrease in the bogie’s fatigue life. Thus, the study of the corrosion behavior of a bogie and its welded joints has the greatest significance for the safe reliability of high-speed trains.

Weathering steel can be used for the bogies of high-speed trains. Cheng et al. [6] investigated the influence of Ni on the corrosion behavior of Q415NH weathering steel by simulating a marine atmosphere, and found that the addition of Ni not only resulted in a positive shift in corrosion potentials but was also helpful in enhancing corrosion resistance. The research suggested that MnCuP weathering steel exhibited high corrosion resistance in simulated coastal, industrial, and coastal-industrial atmospheric environments [7]. Zhu et al. investigated the corrosion mechanism of weathering steel in a simulated industrial atmosphere, and found that the non-metallic oxide affected corrosion resistance [8]. So far, most of the previous studies have focused on the corrosion behavior of these types of steel in different atmospheric environments, and yet there are few reports dealing with the materials used for high-speed train bogies, such as SMA490BW steel [9]. Moreover, the difference in the corrosion behavior between SMA490BW steel and its welded joints is still not clearly understood. In the present work, the corrosion behavior of SMA490BW steel and its welded joints, which are used for high-speed train bogies in China, was investigated using the periodic immersion wet/dry cyclic corrosion test to simulate an industrial atmospheric environment. In order to analyze the corrosion mechanism, corrosion weight loss rate, structure, and composition of rust layers as well as the potentiodynamic polarization curve were also investigated. This provides essential data for the safe reliability of bogies found in high-speed trains in commission.

## 2. Materials and Methods

### 2.1. Materials

The raw material used was SMA490BW steel plate with dimensions of 350 × 150 × 12 mm (length × width × thickness). The welding wire was CHW-55CNH (Sichuan Atlantic Co. Ltd., Zigong, China) with a diameter of 1.2 mm. The shield gas was a mixed gas of 80% argon and 20% carbon dioxide. The chemical compositions of SMA490BW steel and CHW-55CNH welding wire are given in Table 1.

### 2.2. Methods

The SMA490BW steel was polished with abrasive papers, and then cleaned with acetone before welding. The butt joints of SMA490BW steel were prepared by single-wire automatic metal active gas (MAG) arc welding with a TPS5000 welding machine (Fronius Intelligent Equipment China Co. Ltd., Zhuhai, China). The groove type was V-groove with a bevel angle of 60°, and the root face dimension was 1 mm. The process parameters of multipass welding are given in Table 2.

**Figure 1:**
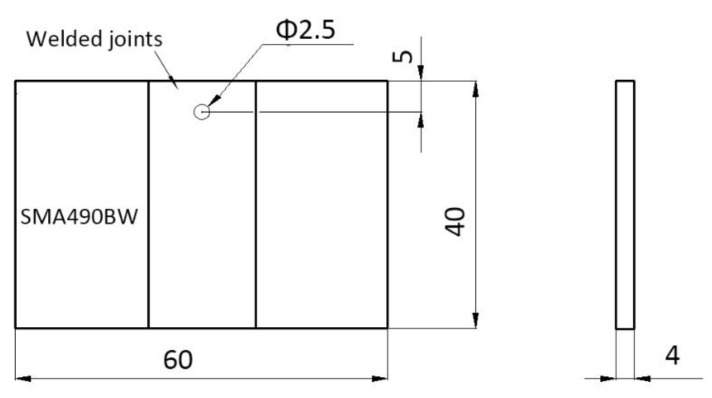
Specimen for the periodic immersion wet/dry cyclic corrosion test.

The periodic immersion wet/dry cyclic corrosion test for SMA490BW steel and its welded joints was carried out on an FL-65 testing machine (Zhongfeijingchi Co. Ltd., Beijing, China) according to ISO 11330: 1999. To simulate an industrial atmospheric environment, the corrosion solution used was NaHSO_3_ with an initial concentration of (1.0 ± 0.05) × 10^−2^mol/L (pH 4.4) and the replenisher was NaHSO_3_ with an initial concentration of (2.0 ± 0.05) × 10^−2^mol/L. The specimen size was 60 × 40 × 4 mm as shown in Figure 1.

First, the specimens were polished by abrasive papers to remove the oxide layer, and then cleaned by means of ultrasonic cleaning with petroleum ether, anhydrous alcohol, and acetone. Second, the initial weight of the specimens after being put in a dryer for 24 h was obtained by electronic scales with an accuracy of 0.1 mg. Third, 25 specimens for each material were put in the testing machine, ready for testing. Each wet/dry cycle test lasted 1 h and contained two stages: (1) the specimens were immersed in NaHSO_3_ (pH 4.4) at a temperature of 45 °C for 12 min; and (2) the specimens were hung and dried at 70% relative humidity for 48 min, and the specimen’s surface temperature was no more than 70 °C. The test period lasted 50 h, 75 h, 100 h, 125 h, and 150 h, respectively. For each material, five specimens were brought out after each test period, and one of them was used for microstructure and phase analysis. The others were rinsed with a HCl solution with hexamethylene tetramine to remove corrosion products, and subsequently washed with distilled water, anhydrous alcohol, and acetone. Then, the specimens were dried for 24 h and weighed.

The corrosion weight loss rate was calculated using the following equation [10]:

(1) $$W=\frac{m_{0}-m_{1}}{2\left(a\times b+b\times c+a\times c\right)t}\times 10^{6},$$

where W is the corrosion weight loss rate (g/m^2^·h), *m*_0_ is the initial weight of the specimens (g), *m*_1_ is the weight of the specimens after the corrosion test (g), *a*, *b*, and *c* are the length, width, and thickness of the specimens, respectively, and t is the corrosion time (h).

The phase composition of the rust layers formed on the specimens after the corrosion test was identified by X-ray diffraction analysis (XRD, Empyrean-X, Almelo, Holland) with Cu-Kα radiation operated at 50 kV and 40 mA with a scanning speed of 2°/min. The surface morphology of the rust layers was observed using a field-emission scanning electron microscope (SEM, Supra 55, Shanghai, China).

The electrochemical test was carried out in a 0.01 mol/L NaHSO_3_ (pH 4.4) electrolyte solution on a CHI600B electrochemical workstation (Shanghai Chenhua instrument Co. Ltd., Shanghai, China) at room temperature. A three-electrode system was adopted, namely, a saturated calomel electrode (SCE) as reference electrode, a Pt electrode as auxiliary electrode, and the specimens with rust layers after the wet/dry cyclic corrosion test as working electrode. The sweeping speed for the potentiodynamic polarization curve was 0.33 mV/s [11].

## 3. Results and Discussion

### 3.1. Corrosion Kinetics

The corrosion weight loss rate of SMA490BW steel and its welded joints in the periodic immersion wet/dry cyclic corrosion test simulating an industrial atmospheric environment was obtained by calculation using Equation (1), and the data are given in Table 3. As shown, a similar pattern was found on SMA490BW steel and its welded joints. The corrosion weight loss rate of SMA490BW steel and its welded joints increased before 75 h and then decreased with increasing corrosion time. In other words, the corrosion process of SMA490BW steel and its welded joints can be divided into two periods, namely, an accelerated corrosion period (before 75 h) and a stable corrosion period (after 75 h). This can be attributed to the change of surface corrosion products during the corrosion process.

**Figure 2:**
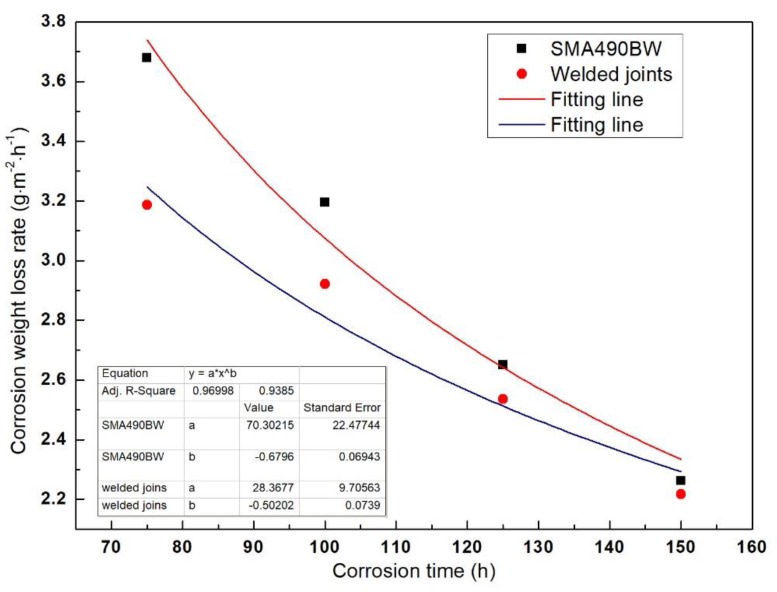
Fitting curve of the specimen’s corrosion weight loss rate as a function of corrosion time.

The corrosion kinetics of steel in atmospheric environment is in accordance with the traditional equation [12] as follows:

(2) $$W=Ct^{n},$$

where *W* is the corrosion weight loss rate, *t* is the corrosion time, and *C* and *n* are constants. The increase in the corrosion weight loss rate in the accelerated corrosion period (before 75 h) can be attributed to surface toughness and work hardening of the specimens, and the specimens’ rust layers did not seem to have a protective capability [13]. Therefore, we only chose the corrosion weight loss rate after 75 h for data fitting in order to verify whether the corrosion kinetics curve was in accordance with Equation (2). Figure 2 shows the fitting curve of the specimens’ corrosion weight loss rate. It can be seen that both correlation coefficients R^2^ are greater than 0.93, which demonstrates that the corrosion behavior of SMA490BW steel and its welded joints should conform to Equation (2). In addition, *n* < 1 means that the corrosion weight loss rate decreases with increasing corrosion time; in other words, the rust layers have a good effect on protecting the substrate. Moreover, the corrosion weight loss rate of the welded joints is lower than that of SMA490BW steel. It can be concluded that the corrosion resistance of the welded joints is higher than that of SMA490BW steel in a simulated industrial atmospheric environment.

### 3.2. Surface Morphology and Phase Analysis of Rust Layers

**Figure 3:**
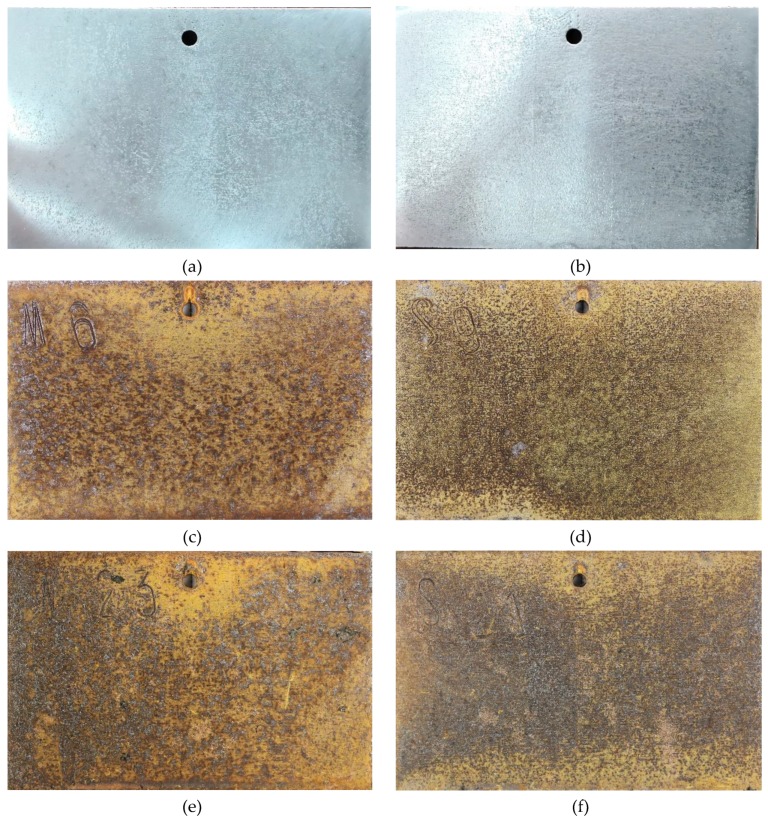
Surface morphology of SMA490BW steel (**a**) as prepared, (**c**) at 75 h, (**e**) at 150 h, and welded joints (**b**) as prepared, (**d**) at 75 h, (**f**) at 150 h, after the periodic immersion wet/dry cyclic corrosion test.

Figure 3 shows the surface morphology of SMA490BW steel and its welded joints after the periodic immersion wet/dry cyclic corrosion test. Obviously, the surface morphology of SMA490BW steel is similar to that of its welded joints. There exists a tawny rust layer on the surface, and a black rust layer is compact and adherent to the substrate [14,15]. Compared with the rust layers of the welded joints after 75 h, the rust layers after 150 h are more compact to the benefit of protecting the substrate and inhibiting the mass transfer process of the corrosion solution. This may explain why the corrosion weight loss rate increased first and then decreased with increasing corrosion time. Furthermore, SMA490BW steel had a looser rust layer in comparison with its welded joints at the same corrosion time, meaning that SMA490BW steel suffered more serious corrosion. These results are in agreement with the corrosion weight loss rate in Table 3.

**Figure 4:**
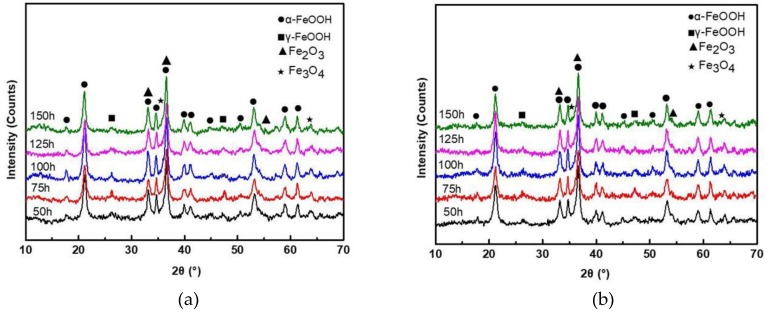
XRD patterns of rust layers formed on (**a**) SMA490BW steel and (**b**) welded joints after the periodic immersion wet/dry cyclic corrosion test.

The XRD patterns of rust layers formed on SMA490BW steel and its welded joints after the periodic immersion wet/dry cyclic corrosion test are shown in Figure 4. It can be seen that the phase compositions of rust layers formed on SMA490BW steel and its welded joints are quite similar. As shown, the rust layers are mainly composed of α-FeOOH, γ-FeOOH, Fe_2_O_3_, and Fe_3_O_4_. In both the rust layers of SMA490BW steel and its welded joints, the diffraction peaks of γ-FeOOH after 150 h are much weaker than those after 75 h. In contrast, the diffraction peaks of α-FeOOH are more intensive after 150 h. To some extent, the XRD results suggest that there exists a transform from porous γ-FeOOH to compact α-FeOOH during the corrosion process [16]. Much research shows that γ-FeOOH is formed from the crystallization of amorphous oxide in the preliminary stage of corrosion and then is partially transformed to α-FeOOH [17,18,19,20]. Furthermore, the volume change due to the phase transformation of γ-FeOOH results in defects in the rust layers, such as pores and cracks. However, α-FeOOH is a stable oxyhydroxide with no phase transformation in the rust layers so that compact and stable rust layers are formed in favor of protecting the substrate. As shown in Figure 4, the diffraction peaks of α-FeOOH of the welded joints are more intensive than that of SMA490BW steel; therefore, the welded joints have a better corrosion resistance than SMA490BW steel.

**Figure 5:**
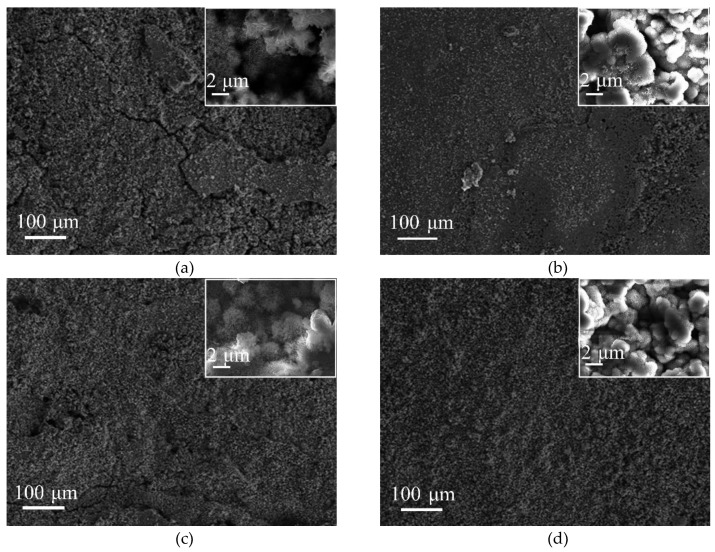
SEM micrographs of the surface rust layers of SMA490BW steel (**a**) 75 h, (**b**) 150 h, and welded joints (**c**) 75 h, (**d**) 150 h, after the periodic immersion wet/dry cyclic corrosion test.

Figure 5 presents SEM micrographs of the surface rust layers of SMA490BW steel and its welded joints after the periodic immersion wet/dry cyclic corrosion test. As shown in Figure 5c, it can be seen that the surface rust layers of the welded joints after 75 h are relatively flat. At the same time, however, a thin layer with small cracks and pores is observed. From the inset in Figure 5c with high magnification, the rust layers consist of flower-like γ-FeOOH and spherical α-FeOOH [21,22]. As shown in Figure 5a, there are more γ-FeOOH and cracks in the rust layers of SMA490BW steel compared with its welded joints. The loose rust layers with porous γ-FeOOH and cracks have no ability to protect the substrate because the porous γ-FeOOH and cracks offer a channel where the corrosion solution can easily invade. Figure 5b,d shows SEM micrographs of the surface rust layers of SMA490BW steel and its welded joints after 150 h, respectively. Although α-FeOOH and γ-FeOOH can be still observed in the rust layers, it is clear that the rust layers of the welded joints after 150 h became much denser than those of SMA490BW steel. It is known that the protective capability of rust layers depends on the compactness and adherence of rust layers formed on the substrate. Consequently, amounts of α-FeOOH and compact inner rust layers in the welded joints provide relatively better protection to the substrate [23]. In other words, the welded joints have a better corrosion resistance than SMA490BW steel under this experimental condition, which is consistent with the corrosion weight loss rate and XRD results presented above.

**Figure 6:**
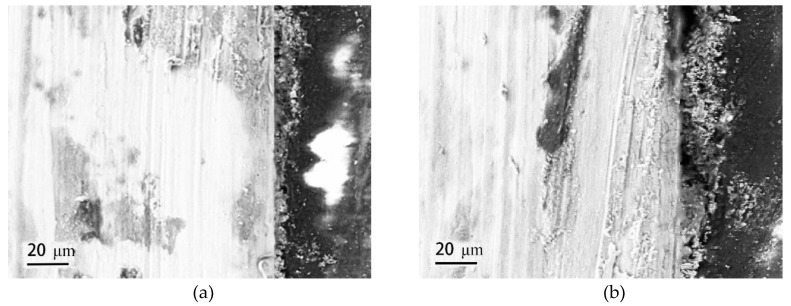

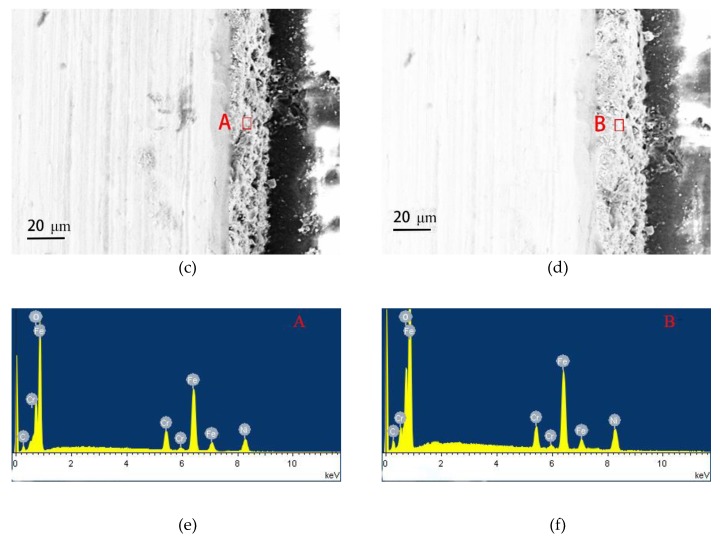
Cross-sectional morphologies of SMA490BW steel (**a**) 75 h, (**c**) 150 h, and (**e**) EDS and welded joints (**b**) 75 h, (**d**) 150 h, and (**f**) EDS after the periodic immersion wet/dry cyclic corrosion test.

Figure 6 gives the cross-sectional morphologies of SMA490BW steel and its welded joints after the periodic immersion wet/dry cyclic corrosion test. As shown, the rust layers of SMA490BW steel and its welded joints after 75 h are thinner and have many cracks. In contrast, the rust layers after 150 h are more compact and more adherent to the substrate. Furthermore, the rust layers of the welded joints are much thicker and more uniform than that of SMA490BW steel. EDS results of the rust layers indicated that alloy elements, such as Ni and Cr, formed in the rust layers after the periodic immersion wet/dry cyclic corrosion test for 150 h.

### 3.3. Electrochemical Test

**Figure 7:**
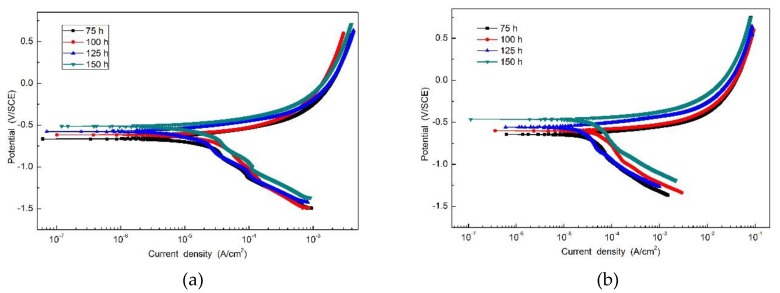
Potentiodynamic polarization curve of (**a**) SMA490BW steel and (**b**) welded joints after the periodic immersion wet/dry cyclic corrosion test.

Figure 7 is the potentiodynamic polarization curve of the specimens with rust layers after the periodic immersion wet/dry cyclic corrosion test. Electrochemical parameters obtained from the polarization curve of SMA490BW steel and its welded joints are given in Table 4. As shown, there is no significant passivation for all the specimens. Moreover, it is obvious that the corrosion potential increases and the corrosion current density decreases with increasing corrosion time, which means that the corrosion rust layers became thicker so that the anodic reaction was restrained by the thick and compact rust layers [24,25]. After 150 h, the welded joints have a higher corrosion potential and lower corrosion current density than SMA490BW steel. These data suggest that the rust layers formed on the welded joints are more protective to the substrate.

### 3.4. Corrosion Mechanism

The atmospheric corrosion of metal is an electrochemical corrosion process that occurs in the thin liquid film in nature. Under conditions of the periodic immersion wet/dry cyclic corrosion test, the surfaces of SMA490BW steel and its welded joints soon lose their metallic luster and begin to corrode. In the wet stage with the presence of oxygen, the anodic oxidation and cathodic reduction reactions in the electrochemical process happen as follows [26]:

(3) $$\mathrm{Anodic}\;\mathrm{reaction}:\;Fe\to Fe^{2+}+2e^{-},$$

(4) $$\mathrm{Cathodic}\;\mathrm{reaction}:\;O_{2}+2H_{2}O+4e^{-}\to 4OH^{-},$$

(5) $$\mathrm{Overall}\;\mathrm{reaction}:\;2Fe+O_{2}+2H_{2}O\to 2Fe{\left(OH\right)}_{2}.$$

Fe(OH)_2_, which is a transient product in the rust, is oxidized to form much amorphous oxide (ferric and ferrous) and Fe(OH)_3_ immediately in the dry stage. Then, γ-FeOOH is formed by crystallization of the amorphous oxide, and transforms afterward to α-FeOOH or Fe_3_O_4_. It is recognized that SO_2_ has the worst effect among air pollutants. A part of SO_2_ adsorbs onto the surface of steel leading to the presence of diffluent FeSO_4_, which is obtained by the chemical reaction between SO_2_, O_2_, and Fe. Furthermore, H_2_SO_4_ is obtained by the further oxidation and strong hydrolysis of FeSO_4_, and then H_2_SO_4_ reacts with Fe [27,28]. For steel, this whole process keeps recurring due to autocatalysis so that much rust is generated from a molecule of SO_2_. In this way, the rusting process is greatly accelerated. However, the corrosion resistance of SMA490BW steel and its welded joints is enhanced due to the addition of alloy elements, such as Ni and Cr. During the early corrosion period, some Ni^2+^ ions replace Fe^2+^ ions, and nanoscale Fe_2_NiO_4_ separates out. Thus, nanoscale Fe_2_NiO_4_ offers enough nucleation centers for the formation of nano-reticular Fe(O,OH)_6_ and inhibits its growth [29,30]. For this reason, a compact, refined and ion-selective rust layer is easy to form. Although corrosion resistance is enhanced with increasing Ni content, there is still a threshold value. Cr can infinitely dissolve in Fe solution so that some Cr^2+^ ions replace Fe^2+^ ions, and this leads to the formation of Cr_x_Fe_1-x_OOH [31]. The rust layers become ion selective due to Cr_x_Fe_1-x_OOH and prevent sulfate ions from permeating effectively [32,33,34]. Furthermore, the enrichment of Cr in defects and grain boundaries has a positive effect on the formation of more compact rust layers. Consequently, the alloy elements have a direct influence on the corrosion resistance of SMA490BW steel and its welded joints.

To explain the difference of corrosion resistance, the main chemical composition of SMA490BW steel and its welded joints before and after the periodic immersion wet/dry cyclic corrosion test were analyzed by EDS and are given in Table 5. Compared with SMA490BW steel, the content of Ni as well as the content of Cr in the welded joints are higher because of the welding wire with a higher content of Ni and Cr. Moreover, the rust layers of the welded joints after 150 h still have a higher content of Ni and Cr than that of SMA490BW steel. As mentioned above, Ni and Cr elements have a positive effect on enhancing corrosion resistance. Therefore, the automatic MAG welded joints exhibit better corrosion resistance than SMA490BW steel because of the higher content of alloy elements.

## 4. Conclusions

The corrosion behavior of SMA490BW steel and its welded joints used for high-speed train bogies was investigated using the periodic immersion wet/dry cyclic corrosion test to simulate an industrial atmospheric environment. The main results are as follows.

The corrosion weight loss rate of SMA490BW steel and its welded joints decreases with increasing corrosion time and then becomes stable because the rust layers after 150 h have a good effect on protecting the substrate.

The alloy elements Ni and Cr accelerate the formation of protective rust layers and are beneficial in enhancing corrosion resistance.

The corrosion weight loss rate, XRD results, SEM micrographs, EDS results, thickness of rust layers, and electrochemical parameters indicate that the welded joints have a better corrosion resistance compared with SMA490BW steel in a simulated industrial atmospheric environment. The main reason is that the content of Ni and Cr in the welded joints is higher than that in SMA490BW steel.

## Figures and Tables

**Table 1:** Chemical composition of experimental materials (wt %).

Materials	C	Si	Mn	S	P	Cu	Cr	Ni	Fe
SMA490BW	≤0.18	0.15–0.65	≤1.40	≤0.005	≤0.035	0.30–0.50	0.45–0.75	0.08–0.25	Bal.
CHW-55CNH	≤0.10	0.35–0.65	1.20–1.60	≤0.025	≤0.025	0.20–0.50	0.30–0.90	0.20–0.60	Bal.

**Table 2:** Welding process parameters.

Number of Weld Passes	Welding Current (*I*)/A	Arc Voltage (*U*)*/*V	Welding Speed (*v*)*/*(mm·s^−1^)	Welding Heat Input (*E*)*/*(kJ·cm^−1^)
1	240	25	8.3	4.3
2–4	245–250	27	5.0–8.3	6.4

**Table 3:** Corrosion weight loss of the specimens.

Material	Corrosion Rate of Weight Loss (*W*)/(g·m^−2^·h^−1^)				
	50 h	75 h	100 h	125 h	150 h
SMA490BW	3.3212	3.6792	3.1943	2.6497	2.2619
Welded joint	3.0149	3.1862	2.9217	2.5361	2.2175

**Table 4:** Electrochemical parameters obtained from the polarization curve of SMA490BW steel and its welded joints.

Corrosion Time	SMA490BW		Welded Joints	
	*E*_corr_(V/SCE)	*i*_corr_(×10^−5^A/cm^2^)	*E*_corr_(V/SCE)	*i*_corr_(×10^−5^A/cm^2^)
75 h	−0.553	1.749	−0.489	1.654
100 h	−0.644	1.411	−0.635	1.393
125 h	−0.716	1.187	−0.698	1.135
150 h	−0.806	1.063	−0.782	0.962

**Table 5:** Main chemical composition of SMA490BW steel and its welded joints before and after the periodic immersion wet/dry cyclic corrosion test (wt %).

Materials	Fe	O	Ni	Cu	Cr	S
SMA490BW (before)	92.9	-	0.25	0.32	0.54	-
SMA490BW (after)	58.51	34.95	0.17	0.15	1.26	1.39
Welded joints (before)	89.94	-	0.50	0.36	0.75	-
Welded joints (after)	56.21	36.21	0.29	0.22	1.81	1.04
